# Thermoelectric Properties of Bi_2_Te_3_: CuI and the Effect of Its Doping with Pb Atoms

**DOI:** 10.3390/ma10111235

**Published:** 2017-10-26

**Authors:** Mi-Kyung Han, Yingshi Jin, Da-Hee Lee, Sung-Jin Kim

**Affiliations:** Department of Chemistry and Nano Science, Ewha Womans University, Seoul 120-750, Korea; mikihan@ewha.ac.kr (M.-K.H.); allkdy77@gmail.com (Y.J.); daheelee1@gmail.com (D.-H.L.)

**Keywords:** Bi_2_Te_3_, thermoelectric properties, doping

## Abstract

In order to understand the effect of Pb-CuI co-doping on the thermoelectric performance of Bi_2_Te_3_, *n*-type Bi_2_Te_3_ co-doped with *x* at % CuI and 1/2*x* at % Pb (*x* = 0, 0.01, 0.03, 0.05, 0.07, and 0.10) were prepared via high temperature solid state reaction and consolidated using spark plasma sintering. Electron and thermal transport properties, i.e., electrical conductivity, carrier concentration, Hall mobility, Seebeck coefficient, and thermal conductivity, of CuI-Pb co-doped Bi_2_Te_3_ were measured in the temperature range from 300 K to 523 K, and compared to corresponding *x*% of CuI-doped Bi_2_Te_3_ and undoped Bi_2_Te_3_. The addition of a small amount of Pb significantly decreased the carrier concentration, which could be attributed to the holes from Pb atoms, thus the CuI-Pb co-doped samples show a lower electrical conductivity and a higher Seebeck coefficient when compared to CuI-doped samples with similar *x* values. The incorporation of Pb into CuI-doped Bi_2_Te_3_ rarely changed the power factor because of the trade-off relationship between the electrical conductivity and the Seebeck coefficient. The total thermal conductivity(κ_tot_) of co-doped samples (κ_tot_ ~ 1.4 W/m∙K at 300 K) is slightly lower than that of 1% CuI-doped Bi_2_Te_3_ (κ_tot_ ~ 1.5 W/m∙K at 300 K) and undoped Bi_2_Te_3_ (κ_tot_ ~ 1.6 W/m∙K at 300 K) due to the alloy scattering. The 1% CuI-Pb co-doped Bi_2_Te_3_ sample shows the highest ZT value of 0.96 at 370 K. All data on electrical and thermal transport properties suggest that the thermoelectric properties of Bi_2_Te_3_ and its operating temperature can be controlled by co-doping.

## 1. Introduction

Bismuth telluride (Bi_2_Te_3_) has been the focus of extensive theoretical and experimental studies as a component of materials for thermoelectric (TE) devices, such as solid-state coolers or generators [1,2,3]. The performance of a thermoelectric material in the aforementioned applications is evaluated in terms of a dimensionless figure of merit ZT, which is defined as (S^2^σ/κ)T; where S is the Seebeck coefficient (or thermopower), σ is the electrical conductivity, κ is the thermal conductivity, and T is the temperature [4]. The product (S^2^σ) is called the power factor. A larger ZT leads directly to a higher conversion efficiency. The main challenge lies in the decoupling of the interdependent thermoelectric parameters (S, σ, and κ), which are strongly coupled to the carrier concentration. Commercial TE devices comprise series of *p*- and *n*-type semiconductor pairs.

The ZT values of commercial Bi_2_Te_3_ compounds are about 1.35 for *p*-type and 0.9 for *n*-type materials [5]. The poor performance of *n*-type Bi_2_Te_3_ based materials compared to that of *p*-type materials seriously inflicts a limitation on making it a more efficient TE device. Both *p*-type and *n*-type characteristics of Bi_2_Te_3_ can be controlled depending on the chemical composition. As is well known, *n*-type Bi_2_Te_3_ have been synthesized by making solid solution with Bi_2_Se_3_, or addition of excess tellurium as an electron donor [6,7]. However, the fabrication of *n*-type Bi_2_Te_3_ thermoelectric materials has a number of technical problems, such as controlling the Se content in Bi_2_Te_3_-Bi_2_Se_3_ solid solution is difficult and Te-rich Bi_2_Te_3_ easily decomposes upon heating. Element doping is a more effective approach to enhance the thermoelectric properties of Bi_2_Te_3_-based alloys [8,9,10,11,12]. Among various dopants, Cu or Cu-halide acts as an excellent additive for improvement of thermoelectric performance of *n*-type Bi_2_Te_3_ [13,14,15,16]. Cu atoms can be either an acceptor or a donor depending on their location in the compound. Cu is also known to improve the reproducibility of thermoelectric materials, due to the formation of Cu–Te bond in the van der Waals gaps, which suppress the escape of Te atoms [17]. The Cu-intercalated Bi_2_Te_3_ bulk shows a significantly enhanced ZT of ~1.12 at 300 K [13], which is the highest ZT value reported for *n*-type Bi_2_Te_3_ binary material. Cu addition can also prevent the oxidation of the Bi_2_Te_3_ [17]. However, the thermoelectric properties of these alloys change with aging time [18]. Studies of the structure and properties of crystals with a co-dopant with Cu content were carried out [16,19,20]. Cu and I atoms co-doped Bi_2_Te_3_ was prepared using the Bridgman method for the improvement of its corresponding thermoelectric properties, whereby the power factor was improved through the co-doping effect of Cu and I, while its thermal conductivity was reduced by forming dispersed Cu-rich nanoprecipitates. The maximum ZT of ~1.16 was achieved at a temperature of 368 K for (CuI)_0.01_Bi_2_Te_3_ [16]. Moreover, various dopants (Au, Mn, Co, Ni, Zn, Ge, Ag, In, Sc, Ti, V, and Sn) in Cu_0.008_Bi_2_Te_2.7_Se_0.3_ have been studied [19,20]. The addition of dopant atoms at Bi sites in *n*-type Cu-intercalated Bi_2_Te_3_ changes the electronic band structure, such as band position and band degeneracies, resulting in an increase of the Seebeck coefficient. As a consequence, peak *ZT* values of 0.88 at 360 K and 0.91 at 320 K were obtained for V-doped and Au-doped Cu_0.008_Bi_1.98_Te_2.7_Se_0.3_, respectively [19,20]. Therefore, it has great potential to further improve the ZT value of *n*-type Bi_2_Te_3_ based materials via compositional tuning approach by adjusting Cu contents or element doping.

In the present study, CuI-Pb co-doped Bi_2_Te_3_ samples were prepared using high temperature solid state reaction method and were consolidated by spark plasma sintering (SPS). The Pb-addition effects on the crystal lattice, the charge transport, and the thermoelectric properties of CuI-doped Bi_2_Te_3_ were evaluated.

## 2. Results and Discussion

**Figure 1:**
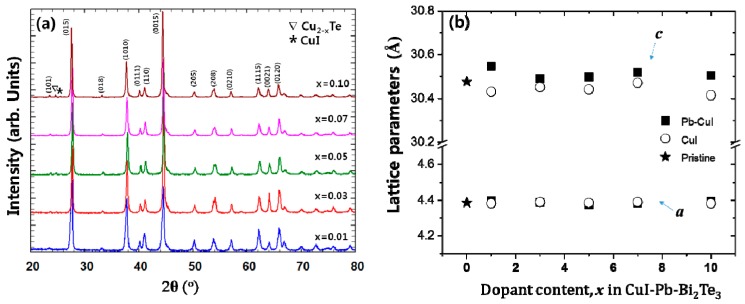
(**a**) Powder X-ray diffraction (XRD) patterns of samples of *x*% CuI-Pb co-doped Bi_2_Te_3_ (*x* = 0.01, 0.03, 0.05, 0.07, and 0.10) with peaks of impurity phases (marked by symbols (∇ and *)); (**b**) Lattice parameters of samples of *x*% CuI-Pb co-doped Bi_2_Te_3_ (*x* = 0, 0.01, 0.03, 0.05, 0.07, and 0.10).

Powder X-ray diffraction (PXRD) patterns of *x*% CuI-Pb co-doped Bi_2_Te_3_ (*x* = 0.01, 0.03, 0.05, 0.07, and 0.10) samples are shown in Figure 1a. As a comparison, undoped Bi_2_Te_3_ and *x*% CuI-doped Bi_2_Te_3_ were prepared under the same synthetic conditions. All of the diffraction peaks are indexed to rhombohedral Bi_2_Te_3_ structure with the space group of R3m (JCPDS, No. 15-0863) [21], with no indication for the existence of a second phase for samples with up to 7% of dopant concentration. Trace amounts of possible impurities including Cu_2−x_Te, and CuI were detected in the 10% CuI-Pb co-doped Bi_2_Te_3_ samples. This result implies that the solubility limit of CuI and Pb dopants in Bi_2_Te_3_ was x < 0.1. While in a previous report, the impurity phase was observed in less than 5% in CuI-doped Bi_2_Te_3_ sample [16], when Pb atoms are co-doped with CuI in Bi_2_Te_3_, the impurity phase was observed only in a 10% CuI-Pb co-doped Bi_2_Te_3_ sample. This result indicates that the solubility of CuI in Bi_2_Te_3_ is increased by addition of Pb atoms. Figure 1b shows the lattice parameters of CuI-Pb co-doped and CuI-doped Bi_2_Te_3_ samples as a function of the dopant fraction. In all of the samples, the in-plane parameter *a* remains constant, while the unit cell parameter *c* along the stacking direction expands with an increasing Pb content in the CuI-Bi_2_Te_3_ system. The result is presumably a consequence of Cu atoms entering into the interstitial site, which increases the distance between the van der Waals layers [13]. A comparison of the covalent radius of Pb (r_Pb_ = 0.147 nm) with that of Bi (r_Bi_ = 0.146 nm) shows that the size of Pb is very close to that of Bi, and thus the ability of Pb atoms for the substitution of Bi atoms in Bi_2_Te_3_ should not be neglected. Halogen atoms such as I (r_I_ = 0.220 nm) are believed to occupy Te (r_Te_ = 0.221 nm) sites in the lattice [22]. The incorporation of iodine atoms to Te sites and Pb atoms to Bi sites drive the changes in bonding parameters. The substituted atoms can bridge two neighboring quintuple layers, thus weakening the interface scattering. Such an analysis exceeds the scope of this paper and would demand quantum chemical calculations of bonding parameters, which will be the aim of our next work.

In our previous work, we demonstrated that doping of Bi_2_Te_3_ samples with 1% CuI enhanced ZT [16]. Thus, we selected 1% CuI-doped Bi_2_Te_3_ sample as a reference material to demonstrate the effect of CuI-Pb co-doping on the charge transport properties. The charge transport properties of 1% CuI-Pb co-doping Bi_2_Te_3_ at room temperature are investigated by Hall effect analysis and are compared to those of 1% CuI-doped Bi_2_Te_3_ and undoped Bi_2_Te_3_. Assuming one carrier type and parabolic bands in our analysis, the carrier concentration (*n*) was calculated from the room temperature (i.e., well within a single-carrier dominated transport) Hall constants using the relationship *R*_H_ = 1/*ne*, where *R*_H_ is the Hall coefficient, *n* is the carrier concentration, and *e* is the electronic charge. The Hall coefficients of specimens are negative, indicating *n*-type conductions. By incorporating Pb in CuI- Bi_2_Te_3_ system, the *n*_e_ value of the bulk samples decreases from ~7.8 × 10^19^/cm^3^ (CuI-doped Bi_2_Te_3_) to ~3.6 × 10^19^/cm^3^ (CuI-Pb co-doped Bi_2_Te_3_), and the corresponding mobility value increases from ~164.6 cm^2^/V∙s to ~216.9 cm^2^/V∙s at 300 K. In comparison, the undoped Bi_2_Te_3_ sample shows the *n* value of ~1.2 × 10^19^/cm^3^and the mobility of 354.9 cm^2^/V∙s at 300 K. This result verifies that the addition of a small amount of Pb significantly decreases the carrier concentration, which should be attributed to the holes generated by the Pb atoms. This demonstrates that facile control of electron concentration can be easily realized by adding Pb atoms to CuI-doped Bi_2_Te_3_ system, yielding an optimal electron concentration of 3−4.5 × 10^19^/cm^3^.

**Figure 2:**
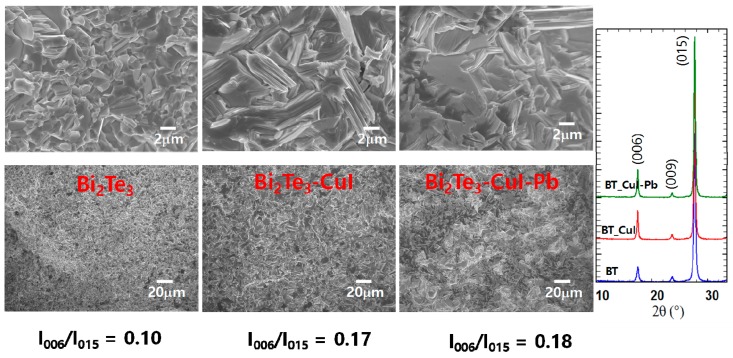
SEM images, and XRD patterns of undoped Bi_2_Te_3_, 1% CuI-doped Bi_2_Te_3_, and 1% CuI-Pb co-doped Bi_2_Te_3_.

Figure 2a shows SEM images of the fractured surfaces of SPSed undoped Bi_2_Te_3_, 1% CuI-doped Bi_2_Te_3_, and 1% CuI-Pb co-doped Bi_2_Te_3_. All of the samples exhibit lamellar structures at the micron scale and no obvious large-scale preferred orientation. The microstructures are dense (>98% of the theoretical density of *n*-type Bi_2_Te_3_ (7.86 g/cm^3^) showing densities of 7.73 g/cm^3^, 7.77 g/cm^3^, and 7.82 g/cm^3^ for undoped Bi_2_Te_3_, 1% CuI-doped Bi_2_Te_3_, and 1% CuI-Pb co-doped Bi_2_Te_3_, respectively. The orientation degree of the (0 0 l) planes, termed as *F*, was calculated with the Lotgering method [23]. In this method, *F* is expressed as the following equations: *F* = *P* − *P*_0_/1 − *P*_0_, *P*_0_ = *I*_0(0 0 *l*)_/∑*I*_0(*h k l*)_, *P* = *I*_(0 0 *l*)_/∑*I*_(*h k l*)_, where *I*_0(0 0 *l*)_ is the intensity of (0 0 *l*) peaks and ∑*I*_0(*h k l*)_ is the sum of intensities of all the peaks for the powders with random orientation; *I*_(0 0 *l*)_ is the (0 0 *l*) peak intensity and ∑*I*_(*h k l*)_ is the sum of the intensities of all peaks for the measured section. We calculated the ratios *I*_(0015)_/*I*_(015)_ of the integrated intensity of (0015) to (015), and represented them in Figure 2b to evaluate the grain orientation anisotropy. All of the samples show anisotropy in the crystal structure; however, the degree of anisotropic orientation is not significant in SPS consolidated polycrystalline samples. The *I*_(0015)_/*I*_(015)_ value for 1% CuI-doped Bi_2_Te_3_ and 1% CuI-Pb co-doped Bi_2_Te_3_ (17–18%) is slightly higher than those for undoped Bi_2_Te_3_ (10%). This indicates that the *c*-axis of the grains after SPS was preferentially oriented parallel to the pressing direction. This result is consistent with a previous report [16], showing the strengthening of the two adjacent quintuple layers by substituting Te with I atoms. Effect caused by sample density or sample orientation is negligible since the relative densities and orientation degree determined by the Lotgering method for CuI-doped and CuI-Pb co-doped Bi_2_Te_3_ samples are nearly same.

**Figure 3:**
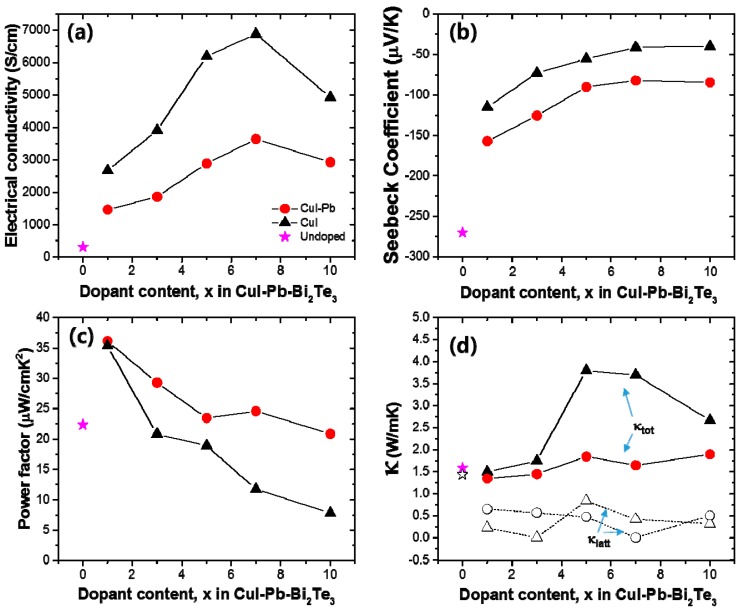
(**a**) Electrical conductivity (σ); (**b**) Seebeck coefficient (*S*); (**c**) power factor; and (**d**) thermal conductivity as a function of dopant concentration *x* in the CuI-doped (black) and CuI-Pb co-doped Bi_2_Te_3_ (*x* = 0, 0.01, 0.03, 0.05, 0.07, and 0.10) system (red) at room temperature.

The thermoelectric properties depend on the dopants (here, we use CuI only and CuI-Pb), dopant content, and temperature. In order to elucidate the effect of dopants and their contents on the thermoelectric properties, the electrical conductivity (σ), Seebeck coefficient (*S*), and power factor of CuI-doped and CuI-Pb co-doped Bi_2_Te_3_ (*x* = 0, 0.01, 0.03, 0.05, 0.07, and 0.10) system as a function of composition was investigated at room temperature, as shown in Figure 3. In both series, with increasing dopant concentration, the electrical conductivity increase, while the Seebeck coefficient decreases simultaneously for up to 7% of dopant concentration. The room temperature electrical conductivity of the undoped Bi_2_Te_3_ (~307 S/cm) is increased by CuI-doping (1% of CuI-doped Bi_2_Te_3_ sample gave ~2673 S/cm). The room temperature electrical conductivity of 1% CuI-Pb co-doped Bi_2_Te_3_ at 300 K was about ~1462 S/cm. This value is significantly lower than that of 1% CuI-doped Bi_2_Te_3_. As shown in Figure 3a, the CuI-Pb co-doped samples show a lower electrical conductivity than that of CuI-doped samples with similar *x* values, confirming the role of Pb as an acceptor [24]. In Figure 3b, the Seebeck coefficients at room temperature were plotted as a function of dopant contents. The value of Seebeck coefficient at 300 K for CuI-doped and CuI-Pb co-doped Bi_2_Te_3_ are about −115 μV/K and −157 μV/K, respectively, while that for undoped Bi_2_Te_3_ is −270 μV/K, which compares well with the previous reported value for *n*-type Bi_2_Te_3_ [2]. The Seebeck coefficients of the CuI-Pb co-doped bulk samples are observed to be higher than that of the CuI-doped sample due to lower carrier concentrations. Normally, Bi_2_Te_3_ shows *p*-type character, however the undoped Bi_2_Te_3_ in this study show *n*-type character. We assume that these differences may arise from the different experimental conditions used for the preparation of undoped Bi_2_Te_3_ crystals. The Bi_2_Te_3_ prepared by the Bridgman method is a *p*-type conductor due to the antisite defect of Bi_Te_. However, in this work, the SPS pressed Bi_2_Te_3_ samples show *n*-type characteristics, which arises from the Te vacancy at the interface. This decrease in electrical conductivity and the increase in Seebeck coefficient in co-doped samples can be explained by an increased carrier scattering related to the incorporation of Pb atoms in the CuI-doped lattice and by decreased carrier concentrations caused by Pb atoms, which act as electron acceptors [24]. As shown in Figure 3c, the CuI-Pb co-doped samples show higher power factors than CuI-doped samples with similar *x* values. The power factors decrease with increasing dopant concentrations. The maximum values of the power factors were observed at *x* = 0.01 for both CuI and CuI-Pb co-doped samples. The benefit of Pb incorporation into CuI-doped Bi_2_Te_3_ was not observed in the power factor because of the trade-off relationship between the Seebeck coefficient and the electrical conductivity. (~35 μW/cm∙ K^2^ for 1% CuI-doped Bi_2_Te_3_; ∼36 μW/cm∙K^2^ for 1% CuI-Pb co-doped Bi_2_Te_3_). This corresponds to an >80% enhancement over the typical value of undoped Bi_2_Te_3_ (22 μW/cm∙K^2^). Figure 3d represents thermal conductivities (closed symbols for κ_tot_ and open symbols for κ_latt_) as a function of dopant content. As the dopant concentration increased, the total conductivity of CuI-doped and CuI-Pb co-doped Bi_2_Te_3_ increased due to the increase of the electronic contribution. The total thermal conductivity κ_tot_ of 1% CuI-Pb co-doped samples (κ_tot_ ~ 1.4 W/m∙K at 300 K) is slightly lower than that of 1% CuI-doped Bi_2_Te_3_ (κ_tot_ ~ 1.5 W/m∙K at 300 K) and undoped Bi_2_Te_3_ (κ_tot_ ~ 1.6 W/m∙K at 300 K) due to alloy scattering. The lattice part (κ_latt_) of the thermal conductivity can be estimated by subtracting the electronic component (κ_elec_) from the measured total thermal conductivity, κ_latt_ = κ_tot_ − κ_elec_. The electronic component is given by the Wiedemann-Franz relation, κ_elec_ = *L*σT, where *L* is the Lorenz number. L is taken to be 1.5 × 10^−8^ V^2^/K^2^ for near-degenerate or degenerate semiconductor [25,26]. The lattice thermal conductivity of 1% CuI-Pb co-doped Bi_2_Te_3_ was 0.66 W/m∙K at 300 K. In contrast to the behavior of κ_tot_ upon increasing the dopant concentration, κ_latt_ slightly decreased with increasing dopant concentration. This result demonstrates clearly that the lattice κ_latt_ is reduced by Pb incorporation through the alloy phonon scattering.

**Figure 4:**
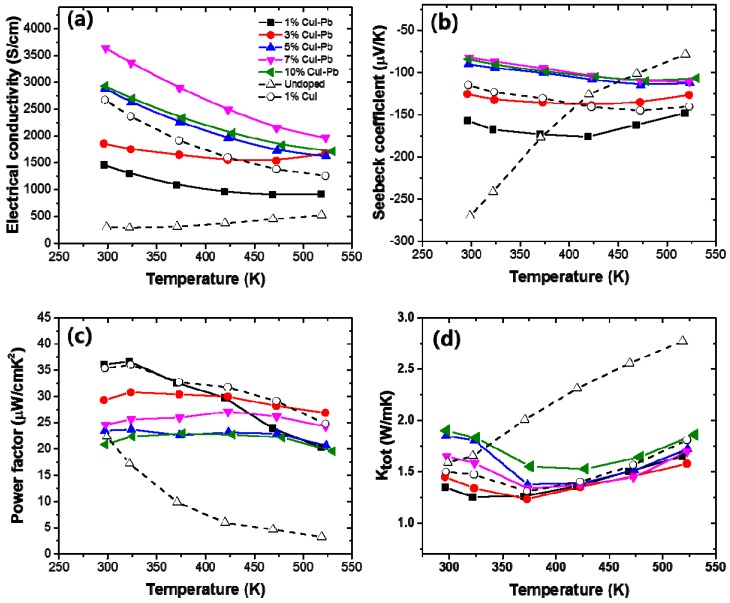
The temperature dependence of (**a**) electrical conductivity (σ); (**b**) Seebeck coefficient (*S*); (**c**) power factor, and (**d**) thermal conductivity of *x*% CuI-Pb co-doped Bi_2_Te_3_ (*x* = 0.01, 0.03, 0.05, 0.07, and 0.10).

Figure 4 shows the electrical transport properties as a function of measured temperature of *x*% CuI-Pb co-doped Bi_2_Te_3_ (*x* = 0.01, 0.03, 0.05, 0.07, and 0.10), when compared with 1% CuI-doped Bi_2_Te_3_ and undoped Bi_2_Te_3_. For all of the samples, a monotonic decrease in electrical conductivity with increasing temperature is observed (Figure 4a), which is indicative of heavily degenerated doping. The variation of the Seebeck coefficient is similar to that of the electrical conductivity, as shown in Figure 4b. The Seebeck coefficient is negative in the whole temperature range, indicating that the majority of charge carriers are electrons (*n*-type). The magnitude of the Seebeck coefficient initially increases and reaches a maximum that is strongly depend on the Pb content *x*. The onset of intrinsic conduction (the maxima of the curves) in these samples shifts to a higher temperature with an increasing dopant content. While the *x* = 0% sample has its maxima at ~300 K, the 1% and 3% sample have their maximum at ~425 K, and the *x* > 5% sample at ~525 K. The maximum value of the Seebeck coefficient (~−176 μV/K) was observed at *x* = 0.01 CuI-Pb content at 425 K. Figure 4c shows the power factors (*S*^2^σ) values as a function of temperature. In this system, the power factor values for the 1% CuI-Pb co-doped ranged from 36 μW/cm·K^2^ at 300 K to 20 μW/cm·K^2^ at 523 K. The CuI-Pb co-doped sample with *x* > 0.03 shows a mild temperature dependence. Figure 4d shows the temperature dependence of the total thermal conductivity κ_tot_ of the samples. The κ_tot_ of all the doped samples firstly decreases due to the increasing phonon-phonon scattering, and then increases when upon further increase of the testing temperature due to the increase of ambipolar thermal contributions arising from the diffusion of electron-hole pairs with the onset of intrinsic contribution [27].

**Figure 5:**
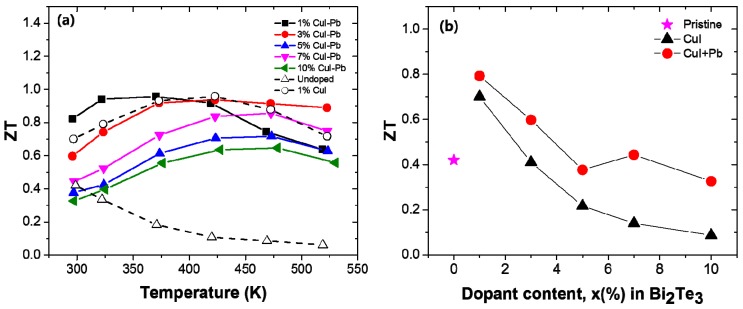
(**a**) Temperature dependency; and (**b**) Dopant content dependency of the room temperature ZT for *x*% CuI-Pb co-doped Bi_2_Te_3_ (*x* = 0.01, 0.03, 0.05, 0.07, and 0.10), 1% CuI-doped Bi_2_Te_3_, and undoped Bi_2_Te_3_.

The dimensionless figure of merit ZT of the samples with different dopant concentration (*x*) are shown in Figure 5a as a function of temperature. The magnitude of the ZT initially increases and reaches a maximum that is strongly dependent on the dopant content *x*. When the temperature is above ~400 K, the ZT values decrease due to the appearance of intrinsic excitation at a higher temperature. In this experiment, the ZT_max_ of the 1% CuI-Pb co-doped sample was about 0.96 at 370 K, while the highest value ZT_max_ was about 0.96 at 422 K for the 1% CuI-doped sample. The incorporation of Pb into CuI-doped Bi_2_Te_3_ led to a shift of the peak position of ZT_max_ to lower temperatures. This result shows that the optimization of the operating temperature can be controlled by co-doping. For practical applications of thermoelectric materials, the ZT values at room temperature are also important. Figure 5b shows the room temperature ZT of the samples as a function of the dopant concentration. The undoped Bi_2_Te_3_ sample shows a low ZT of ~0.42 at 300 K due to its very poor electrical properties. The highest ZT of 0.79 and 0.70 at 300 K were achieved for the 1% CuI-Pb doped sample and 1% Cu-doped Bi_2_Te_3_ sample, respectively, which are both significantly improved when compared with those of the undoped sample. All of the evidences about electrical and thermal transport properties suggest that the *n*-type ZT of Bi_2_Te_3_ can be enhanced by the incorporation of Pb with CuI dopant. Further improvement in its TE properties can be expected by choosing suitable combination of dopants.

## 3. Materials and Methods

### 3.1. Synthesis of Bulk ingot and Powder Processing

*n*-type Bi_2_Te_3_ co-doped with *x* at % CuI and 1/2*x* at % Pb (*x* = 0, 0.01, 0.03, 0.05, 0.07, and 0.10) were prepared by means of the conventional high-temperature solid-state reaction method, using Bi, Te, CuI and Pb (All 99.999%, from Alfa Aesar, Ward Hill, MA, USA) as starting materials. For convenience, the samples are labeled as dopant contents, such as that Bi_2_Te_3_ + *x* CuI + 1/2*x* Pb with *x* is labeled as *x*% CuI-Pb co-doped Bi_2_Te_3_. *n*-type Bi_2_Te_3_ doped with *x* at % CuI alone was prepared under identical experimental conditions for comparison. The corresponding elements were sealed in appropriate ratio in evacuated fused silica tubes (14 mm diameter, 1 mm wall thickness) under a residual pressure of ~10^−4^ Torr. The sealed tubes were heated to 1000 °C over 12 h, and then held at 1000 °C for 12 h while rocking the liquid to facilitate a complete mixing of the contents. The tubes were slowly cooled to 800 °C over a period of 12 h and then quenched to room temperature. The cast ingot samples were powdered by ball milling in an Ar-filled glove box and the ground powder was passed through a 53 μm-mesh sieve. To obtain dense bulk samples, spark plasma sintering (SPS) was performed under Ar atmosphere by using SPS machine (SPS-211Lx, Fuji Electronic Industrial Co., Ltd., Osaka, Japan). Typically 12–13 g of the powdered samples were loaded into the graphite die with an inside diameter of 14 mm and heated to 425 °C for 5 min at a heating rate of 100 °C/min and held there for 5 min under an axial pressure of 50 MPa under a vacuum of 1.4 × 10^−2^ Torr.

### 3.2. Characterization of Materials

Powder diffraction pattern was obtained with a Rigaku D/MAX X-ray (Rigaku Co., Shibuya-Ku, Tokyo, Japan, 40 kV and 30 mA) diffractometer with CuK_α_ radiation (λ = 1.54056 Å). The lattice parameters were obtained by least squares refinement of data in the 2θ range of 10°~70°, with the assistance of a Rietveld refinement program [28]. The carrier concentration was measured by a Hall measurement system (BIO-RAD, HL5500PC, Milpitas, CA, USA) at room temperature in air. The morphologies and chemical composition of the SPS-sintered samples were investigated via field-emission scanning electron microscopy (FE-SEM, JEOL JSM-5800F, JEOL Ltd., Akishima, Tokyo, Japan).

### 3.3. Characterization of Thermoelectric Properties

In order to investigate the thermoelectric properties, the sample (~13 g) after SPS were cut into rectangular shapes with dimensions of ~3 mm × 3 mm × 10 mm and a disk-shape of about ~14 mm diameter and 2 mm thickness. The former specimens were subjected to the Seebeck coefficient and electrical conductivity measurements (ULVAC-RIKO ZEM-3, ULVAC Inc., Yokohama, Kanagawa, Japan), and the latter to thermal diffusivity measurements using a NETZSCH LFA 457 MicroFlash™ instrument (NETZSCH, Selb, Germany). The thermoelectric properties of the samples were measured along the direction perpendicular and parallel to the SPS pressing direction. Only results of perpendicular direction measurements are shown in the manuscript. The thermal conductivity, κ_tot_, can be obtained from the relationship κ_tot_(T) = D(T)∙*C_p_*(T)∙ρ(T), where *C_p_* is the specific heat, D(T)is the thermal diffusivity, and ρ(T) is the density of the sample. Thermal diffusivity and specific heat were determined by the flash diffusivity-heat capacity method with a Pyroceram standard using the method described in detail in the literature [29]. Sample density (ρ(T)) was calculated from the sample’s geometry and mass. Electrical conductivity and Seebeck coefficient were measured simultaneously under Helium atmosphere from room temperature to approximately 550 K. The Seebeck coefficients were measured three times, with different temperature gradients between 5 and 15 K at each temperature step.

## 4. Conclusions

In this work, utilizing second dopant, we successfully shifted the optimum ZT of an *n*-type Bi_2_Te_3_-based compound towards a lower temperature. This demonstrates that facile control of the electron concentration can be realized by adding Pb atoms to the CuI-doped Bi_2_Te_3_ system, yielding an optimal electron concentration of 3–4.5 × 10^19^/cm^3^. Whereas, the change of room temperature power factor as a consequence of Pb addition was not notable, the thermal conductivity decreased with Pb addition due to the alloying scattering. The maximum ZT of 0.96 was obtained at 370 K for 1% CuI-Pb co-doped Bi_2_Te_3_. In comparison with 1% CuI-doped and undoped Bi_2_Te_3_, the ZT of 1% CuI-Pb co-doped Bi_2_Te_3_ (ZT ~0.79) at room temperature was enhanced by more than 12% and by 88%, respectively.
